# Multilayered Tissue Assemblies Through Tuneable Biodegradable Polyhydroxyalkanoate Polymer (Mesh)-Reinforced Organ-Derived Extracellular Matrix Hydrogels

**DOI:** 10.3390/gels11070539

**Published:** 2025-07-11

**Authors:** Vasilena E. Getova, Alex Pascual, Rene Dijkstra, Magdalena Z. Gładysz, Didi Ubels, Malgorzata K. Wlodarczyk-Biegun, Janette K. Burgess, Jeroen Siebring, Martin C. Harmsen

**Affiliations:** 1 Department of Pathology and Medical Biology, University Medical Center Groningen, University of Groningen, 9713 GZ Groningen, The Netherlands; alex.pascual-cid@dpag.ox.ac.uk (A.P.); j.k.burgess@umcg.nl (J.K.B.); 2 W.J. Kolff Institute for Biomedical Engineering and Materials Science-FB41, University Medical Center Groningen, University of Groningen, 9713 AV Groningen, The Netherlands; 3 Center for Dentistry and Oral Hygiene, University Medical Center Groningen, University of Groningen, 9713 AV Groningen, The Netherlands; r.j.b.dijkstra@umcg.nl; 4 Pharmaceutical Analysis, Groningen Research Institute of Pharmacy, University of Groningen, P.O. Box 196, XB20, 9700 AD Groningen, The Netherlandsm.k.wlodarczyk@rug.nl (M.K.W.-B.); 5 Polymer Science, Zernike Institute for Advanced Materials, University of Groningen, Nijenborgh 4, 9747 AG Groningen, The Netherlands; didiubels@hotmail.com; 6 Biotechnology Centre, Silesian University of Technology, Krzywoustego 8, 44-100 Gliwice, Poland; 7 Groningen Research Institute for Asthma and COPD (GRIAC), University Medical Center, University of Groningen, 9713 AV Groningen, The Netherlands; 8 Research Centre Biobased Economy, Hanze University of Applied Sciences, Zernikeplein 11, 9747 AS Groningen, The Netherlands; j.siebring@pl.hanze.nl

**Keywords:** extracellular matrix, hydrogel, PHBV, P34HB, biodegradation, 3D printing, melt electrowriting

## Abstract

Multi-layer cell constructs produced in vitro are an innovative treatment option to support the growing demand for therapy in regenerative medicine. Our research introduces a novel construct integrating organ-derived decellularised extracellular matrix (dECM) hydrogels and 3D-printed biodegradable polymer meshes composed of poly(3-hydroxybutyrate-co-3-hydroxyvalerate) (PHBV) and poly(3-hydroxybutyrate-co-4-hydroxybutyrate) (P34HB) to support and maintain multiple layers of different cell types. We achieved that by integrating the mechanical stability of PHBV+P34HB, commonly used in the food storage industry, with a dECM hydrogel, which replicates organ stiffness and supports cellular survival and function. The construct was customised by adjusting the fibre arrangement and pore sizes, making it a suitable candidate for a personalised design. We showed that the polymer is degradable after precoating it with PHB depolymerase (PhaZ), with complete degradation achieved in 3–5 days and delayed by adding the hydrogel to 10 days, enabling tuneable degradation for regenerative medicine applications. Finally, as a proof of concept, we composed a three-layered tissue in vitro; each layer represented a different tissue type: epidermal, vascular, and subcutaneous layers. Possible future applications include wound healing and diabetic ulcer paths, personalised drug delivery systems, and personalised tissue implants.

## 1. Introduction

Damage to organs and tissues demands functional repair or regeneration. Upon substantial damage, such as after oxygen deprivation or as a consequence of chronic insults, such as in diabetic ulcers, the body’s repair and regeneration processes are inadequate. In such scenarios, tissue engineering may provide a helping hand. In the case of dermal lesions, covering the chronic wound with a patch that promotes wound healing has been a topic of research for several decades. The skin comprises a multilayered compartment of cellular constituents, each with its own specific extracellular matrix. Cellular layers comprise the epidermis with keratinocytes, the well-vascularized tissue dermis with mesenchymal cells for dermal maintenance, and the sub-dermis with nourishing adipose tissue. These cellular compartments are all held together by an extracellular matrix, which are differently organised for all three layers: compact in the epidermis, collagen-rich in the dermis, and sparse but cell-rich in the sub-dermis [1,2,3].

Our previous research showed that organ-derived decellularised extracellular matrix (dECM) hydrogels from organs such as the heart, aorta, lung, and skin support vascular network formation by endothelial cells in vitro [4,5]. Similarly, these organ-derived ECM hydrogels facilitate the phenotypic maintenance of mesenchymal cell types like fibroblasts and mesenchymal stem cells (MSC) [6]. A layer-by-layer structure, e.g., by combining different hydrogels with representative cells, would appear as a suitable in vitro model to replicate the tissue’s multilayered organisation. Such constructs have been used to replicate various tissues; e.g., constructs manufactured from hydrogels [7], and some implementing a 3D printing technique [8].

Diverse biocompatible polymers and materials are commercially available for clinical use, from the standard-of-practice mesh for repairing hernia, such as polypropylene (PP) and polyethene terephthalate (PET) [9], to newly developed 3D-printed personalised implants for bone defects, such as Ti-6Al-4V alloy [10]. In recent years, bacterially produced products, i.e. natural polyhydroxyalkanoate polymers (PHAs), have received increasing attention. More than 150 different monomeric PHA building blocks have been identified, enabling the optimisation of favourable mechanical properties, biocompatibility, and degradation times [11]. PHAs are biodegradable thermoplastics manufactured from renewable resources, are biocompatible, generally non-toxic, and, therefore, very attractive for biomedical applications. Not only is the production process upscalable to an industrial scale, but it is also cost-efficient [12,13,14]. Additionally, these polymers are enzymatically degradable by bacterially produced depolymerases.

PHBV is highly biocompatible and has food contact material status (allowed for use in food processing and packaging). Its fibres have already been used for biomedical applications in vitro models, such as cardiac patches or nerve tissue grafts [15,16]. On the other hand, P34HB is FDA (U.S. Food and Drug Administration) approved for medical applications, has promising mechanical properties, as it is less brittle than PHBV, and has been researched for biomedical applications in vitro, such as for drug-eluting stents andcartilage regeneration and repair [16,17,18].

Recently, fused deposition modelling (FDM) printable PHAs consisting of poly(3-hydroxybutyrate-co-3-hydroxyvalerate) (PHBV) and poly(3-hydroxybutyrate-co-4-hydroxybutyrate) (P34HB, in the literature also referred to as P3HB-4HB) became available [13]. Printing by melt electrowriting (MEW) has also entered the manufacturing arena [14,19]. MEW is a high-resolution 3D-printing technique that combines electrohydrodynamic jetting and melt extrusion principles, allowing accurate deposition of nano- to micrometre-sized (in diameter) strands of the biocompatible polymer in a layer-by-layer manner [19]. This allows high-precision printing with the flexibility of the design. Throughout this paper, the final biodegradable product used is referred to as either a polymer or mesh, as these two terms are used interchangeably.

In this paper, we argued that a next-generation layered-cell construct could combine cell-loaded dECM hydrogels supported by 3D-printed biodegradable meshes with various pore sizes for each layer. In this proof-of-concept study, we generated triple-layered stacks of cell-laden gels reinforced by meshes composed of a PHBV+P34HB blend and optimised mesh shape and size, cell seeding, and degradation properties for future applications in tissue engineering.

## 2. Results and Discussion

### 2.1. Mesh Characterisation: Pore Size

**Figure 1:**
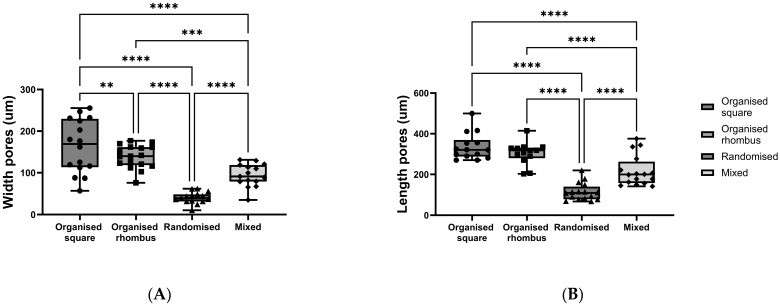
Mesh characterisation: Pore size of the biodegradable material printed with different fibre organisation (width (**A**) and length (**B**)). *p* ≤ 0.01 **; *p* ≤ 0.001 ***; *p* ≤ 0.0001 ****; 2-way ANOVA analysis.

Meshes with four distinctive designs were produced using the PHBV+P34HB polymer blend: (1) organised square, (2) organised rhombus, (3) randomised, and (4) mixed organised/randomised. Pore sizes (width (W) and length (L)) differed between all four polymer designs, with the largest pores in the randomised square polymer mesh (W mean 163.6 ± 64.07 µm SD; L mean 336.5 ± 66.07 µm SD) and the smallest pores in the randomised polymer mesh (W mean 40.43 ± 13.83 µm SD; L mean 114.2 ± 43.07 µm SD) (width *p* < 0.0001; length *p* < 0.0001) (Figure 1A,B).

The printing accuracy and reproducibility were sufficient, given that some fibres misalignment was also observed, as visible in the figure.

### 2.2. Construct Characterisation: Relaxation/Stiffness/Elements

**Figure 2:**
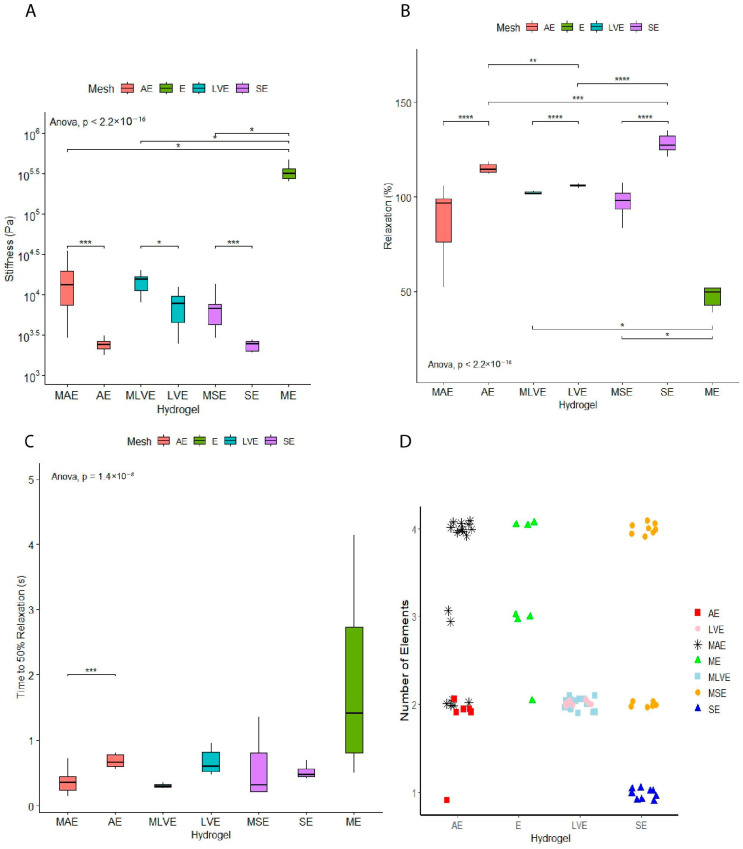
Stiffness measurements. To characterise the construct, stiffness (**A**), stress relaxation (**B**), 50% relaxation (**C**), and the number of elements (**D**) were measured. *p* ≤ 0.05 *; *p* ≤ 0.01 **; *p* ≤ 0.001 ***; *p* ≤ 0.0001 ****; 2-way ANOVA analysis. *(AE—aorta dECM hydrogel with mesh and endothelial cells; E—mesh with endothelial cells; LVE—left ventricle dECM hydrogel with mesh and endothelial cells; SE—skin dECM hydrogel with mesh and endothelial cells).*

A lower stiffness was observed when comparing organ-derived hydrogels to the hydrogel-mesh group. The stiffness did not differ between the different organ-derived hydrogels without a mesh. The mesh with the endothelial cells group (297 ± 138 kPa) showed the highest stiffness compared to the organ-derived hydrogels with a mesh (aorta: 14 ± 9 kPa, left ventricle: 14 ± 4 kPa, skin: 7 ± 3 kPa). (Figure 2A). Therefore, as expected, the groups with meshes led to elevated stiffness values overall.

The stress relaxations of dECM hydrogels differed in an organ-dependent fashion. Skin-derived hydrogels show the highest stress relaxation (128 ± 5%), while left ventricle hydrogels had the lowest stress relaxation (106 ± 0.8%). Stress relaxation of aortic hydrogels (115 ± 3%) was reminiscent of skin hydrogels (128 ± 5%). As expected, reinforcing cell-embedded hydrogels with printed polymer meshes decreased stress relaxation for all three combinations. A mesh loaded with endothelial cells showed the lowest overall relaxation within this study (Figure 2B).

Stress relaxation was over 100% on occasion, likely due to changes in dECM, cells, or both. As in previous studies, a time to 50% relaxation was determined (Figure 2C). No differences were observed, except for an increased time of aortic hydrogels compared to polymer-mesh-reinforced aortic hydrogels (*p* < 0.001).

The organ-derived dECM hydrogels supported with a mesh showed an overall increase in the number of contributing elements to the relaxation process of the hydrogel. Only the left-ventricle-derived dECM hydrogel (2 elements) did not show this increase in the number of contributing elements after adding the mesh (Figure 2D).

### 2.3. Randomised Fibres Proved as Optimal Construct Geometry for Cell Adhesion

**Figure 3:**
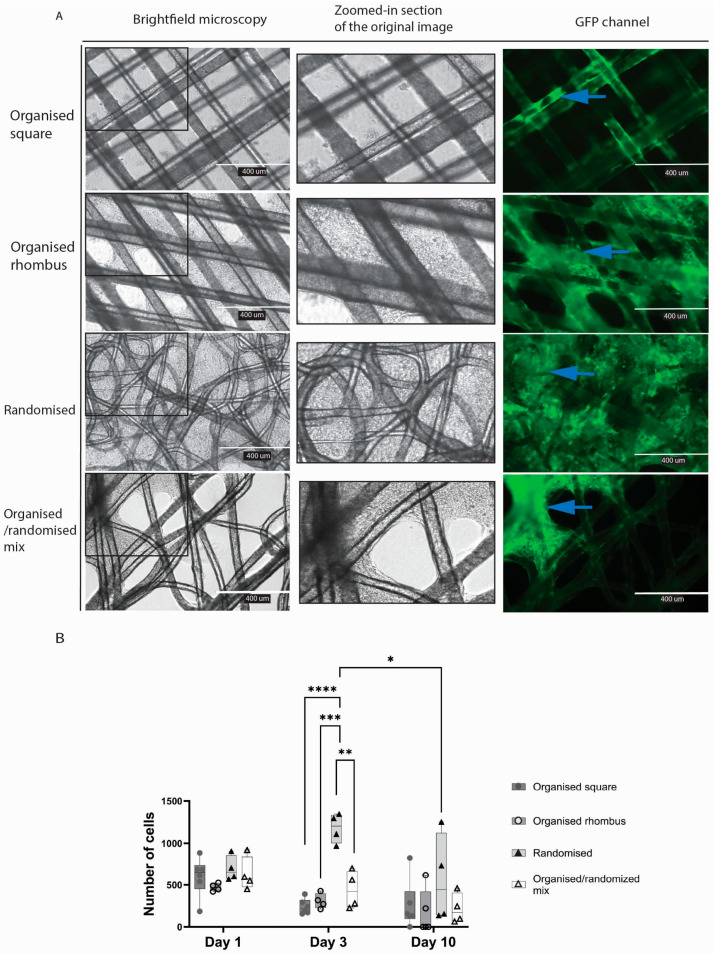
Comparing the biocompatibility of different PHBV+P34HB designs with endothelial cells. Brightfield microscopy, including a zoomed-in section of the original image (**A**) and fluorescence microscopy after live fluorescent staining, with quantitative analysis of the fluorescent staining. Blue arrows point to cells visible in green due to staining (**B**). *p* ≤ 0.05 *; *p* ≤ 0.01 **; *p* ≤ 0.001 ***; *p* ≤ 0.0001 ****; 2-way ANOVA analysis. (*GFP—green fluorescent protein*).

Meshes floating in culture wells appeared to attract endothelial cells that formed sheets of cells in the gaps between the fibres (Figure 3A). In meshes with an organised square architecture, endothelial cells adhered to the fibres but barely spread out to form sheets and cover the pores. In contrast, in the case of the organised rhombus and randomised MEW meshes, the inter-fibre gaps were closed by endothelial sheets after ten days. Cell sheets were formed heterogeneously on organised/randomised meshes. The number of cells was higher in the randomised mesh group compared with two of the other three mesh designs on day three of culture (organised square *p* < 0.0001; organised rhombus *p* = 0.0003; organised/randomised mix *p* = 0.0039) (Figure 3B).

**Figure 4:**
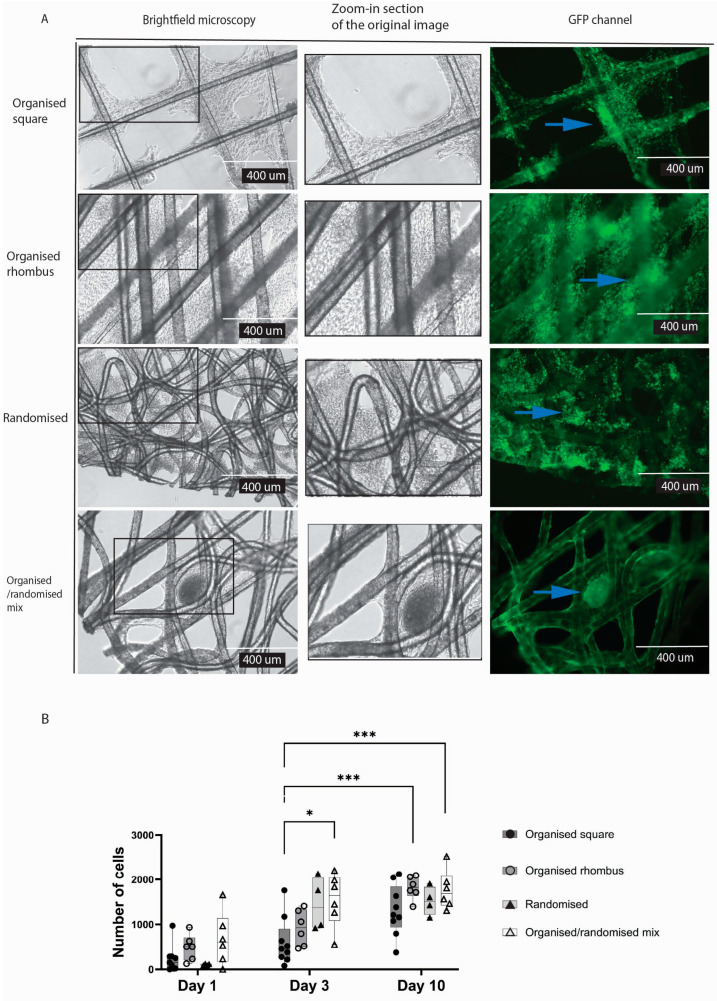
Comparing the biocompatibility of different PHBV+P34HB designs with fibroblasts. Brightfield microscopy, including a zoomed-in section of the original image (**A**) and fluorescence microscopy after live fluorescent staining, with quantitative analysis of the fluorescent staining. Blue arrows point to cells visible in green due to staining (**B**). *p* ≤ 0.05 *; *p* ≤ 0.001 ***; 2-way ANOVA analysis. *(GFP—green fluorescent protein).*

In contrast to endothelial cells, typical connective tissue cells, i.e. fibroblasts, formed cellular sheets in the gaps irrespective of the meshes’ architecture (Figure 4A). Yet, organised square meshes appeared to reduce the formation of fibroblast sheets. The randomised and organised/randomised meshes (compared to organised square design (*p* = 0.0103) were nearly completely filled with cells after three days of culture, while both other architectures were nearly completely filled after 10 days (Figure 4B).

Cell sheets were formed heterogeneously on organised and randomised polymers due to the diverse pore sizes; more cell sheets were formed in the randomised mesh. The fibres of the randomised mesh visibly have more crossing points with different crossing angles, resulting in heterogeneous topography. An essential characteristic of the randomised mesh is its smaller pore size, which provided more closely positioned fibres for cell attachment and support.

Our model combined well-studied biodegradable polymers, PHBV and P34HB, with dECM hydrogels. Both are used in the food container storage industry [20,21] and have been researched in vitro [22,23,24], with limited research in vivo [25]. The clinical use of these PHAs is still on the horizon [24]. Our study demonstrated the biocompatibility of the polymer blend with two diverse cell lines. Randomised fibre design, more fibre crossing points, and smaller pores were vital for endothelial cell adhesion. Endothelial cells represent the lining of our blood vessels; they are sensitive and rapidly respond to external stimuli in their environment. Therefore, their construct must provide enough fibre area for cell adhesion.

### 2.4. dECM Hydrogel Slows Down Degradation Compared to Mesh Alone

**Figure 5:**
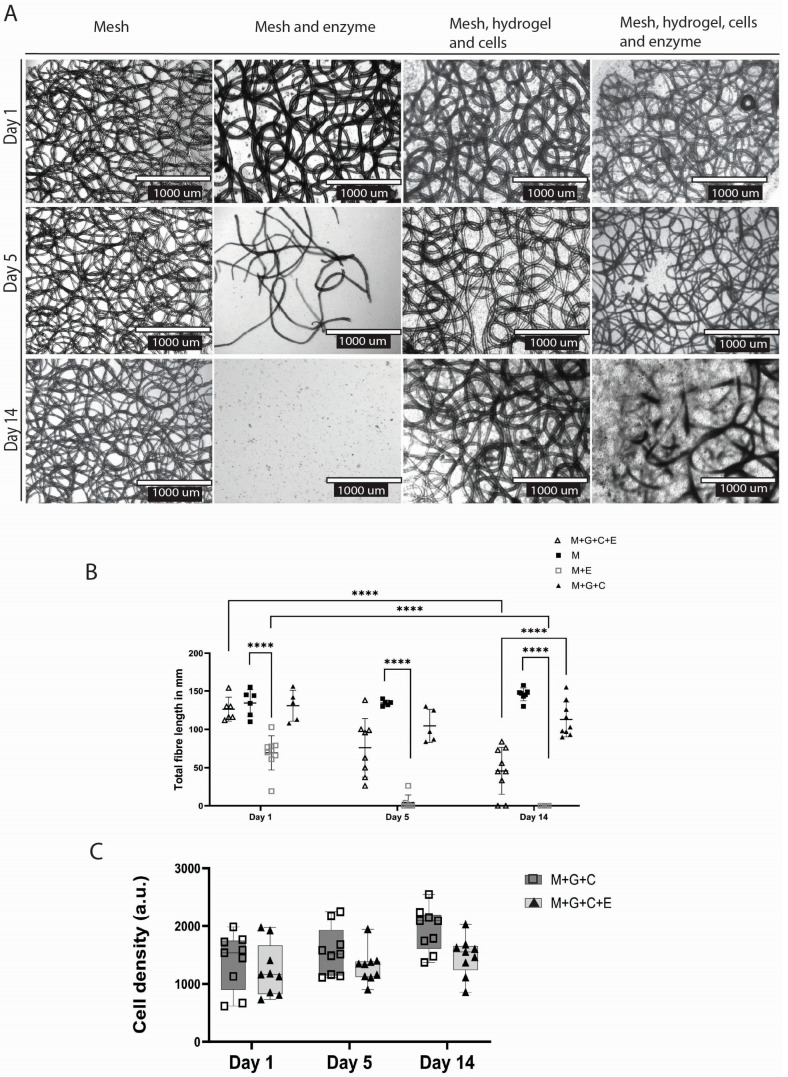
Enzymatic degradation of the constructs over time (**A**), quantitative representation of the degradation measured by the total fibre length in mm (**B**), and quantitative measurements of endothelial cells (**C**). *p* ≤ 0.0001 ****; 2-way ANOVA analysis. *(M—mesh; M+E—mesh and enzyme; M+G+C—mesh, dECM hydrogel and cells; M+G+C+E—mesh, dECM hydrogel, cells and enzyme).*

In all groups treated with the PHB depolymerase enzyme, polymer degradation was observed over time (Figure 5A). In the enzyme and mesh group, compared to the control (no enzyme), total fibre length decreased within one day (*p* < 0.0001) (mean length 69.44 mm ± 22.31 SD); after day five, visible fibre degradation had occurred (mean length 4.386 mm ± 9.642 SD), and at day 14, most of the fibres were degraded (mean length 0.0 mm ± 0.0 SD) (Figure 4B).

At the end of the two weeks, the total fibre length showed a decrease in the mesh + hydrogel + cells group treated with enzyme (mean length 45.78 mm ± 30.77 SD) compared to the control (mean length 113 mm ± 22.72 SD) (*p* < 0.0001). Degradation was slowed in the case of polymer meshes embedded within the dECM hydrogel. In these samples, degradation was visually recorded only from day five, when fibre degradation became apparent. This resulted in a larger standard deviation in this group (SD day one = 15.56; SD day 14 = 30.77) as some polymer fibres started degrading heterogeneously (Figure 5A,B)

### 2.5. Enzymatic Degradation Does Not Hinder Biocompatibility

The number of endothelial cells did not differ between the construct versus the construct with the degradation enzyme at any point: at day 14 (*p* = 0.1503), at day 5 (*p* = 0.7146), and at day 1 (*p* = 0.9705) (Figure 5C). Therefore, the degradation process and pre-coating the polymer with the degradation enzyme did not disturb or negatively affect the cell viability.

We showed that the PHBV+ P34HB polymer blend was completely degraded by the PHB depolymerase enzyme, with pre-incubation, within 3–5 days. The dECM hydrogel slowed down degradation, with partial degradation on day ten. This provides one more factor that regulates the degradation time of the mesh in addition to enzyme concentration. A construct with tuneable degradation has many possible implementations in regenerative medicine, from simple drug delivery platforms to complex internal tissue regeneration patches.

Further research is needed to investigate the correlation between the hydrogel-to-polymer ratio and degradation time, providing exact data for more controlled degradation of the construct.

### 2.6. dECM Hydrogels Inclusion Improved Cell Adhesion and Survival in the Construct (Hydrogel/Mesh)

**Figure 6:**
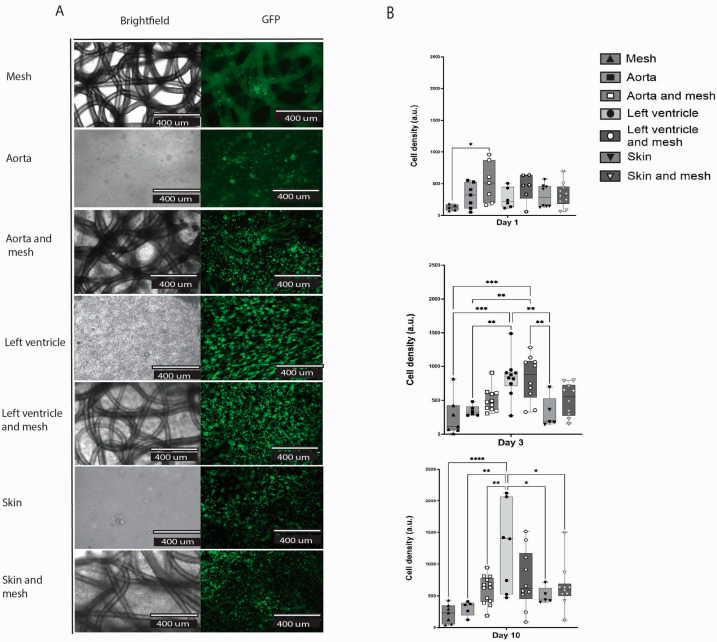
Endothelial cells cultured in the construct: PHBV+P34HB and three different dECM hydrogels, in brightfield images and GFP channel after a live cell staining (**A**). Quantitative representation of cell density at day 1, 5, and 10 of cell culture in the different groups (**B**). *p* ≤ 0.05 *; *p* ≤ 0.01 **; *p* ≤ 0.001 ***; *p* ≤ 0.0001 ****; 2-way ANOVA analysis. *(GFP—green fluorescent protein).*

Here, we compared three groups of hydrogels, where each group was made from a different organ ECM, with and without a mesh: aorta, left ventricle, and skin (Figure 6A). There was no difference in cell count within each separate dECM hydrogel group with and without a mesh (Figure 6B). At day 10, the control group of a mesh and cells showed the lowest cell count of all groups (mean 220.4 ± 150 SD); only the LV hydrogel showed a statistically significant increase in cell number compared to mesh alone (*p* < 0.0001). The most living cells were observed in the LV hydrogel group (mean 1250 ± 692 SD), followed by the LV hydrogel plus mesh group (Mean 756.7 ± 463.8 SD).

The hydrogel was homogeneously spread throughout the mesh and filled all pores. In addition, embedded cells were homogenously distributed through the dECM hydrogels (Figure 6A). Interestingly, the cells do not follow the pattern of the polymer fibres, meaning they did not preferentially attach to the fibres, but rather to the hydrogel, as shown by the homogenous distribution. The cells attach to the mesh fibres in the mesh group alone, likely because this was the only substrate available.

Our results showed better cell attachment and survival in groups with ECM hydrogel compared to groups without. The topography of the extracellular matrix (ECM) provided an in vitro environment that closely resembles the in vivo conditions [26].

### 2.7. Three-Layered Polymeric Constructs for Multi-Cellular Systems

**Figure 7:**
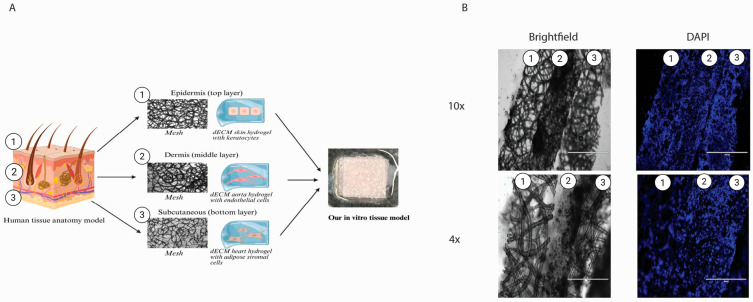
In vitro generation of a skin graft composed of the layers. The first (outside) layer is composed of a dense mesh of dECM skin hydrogels and keratocytes. The second (middle) layer consisted of a mesh with a higher pore size and dECM aorta hydrogel, along with endothelial cells. The last (bottom) layer is comprised of a mesh with dECM heart hydrogel and adipose stromal cells (**A**). Brightfield and DAPI channel microscopic pictures of a cross-section of the construct, with separate layers indicated (**B**). *(DAPI—4′,6-diamidino-2-phenylindole).*

We generated a construct consisting of three layers of in vitro engineered tissue (Figure 7A). The layers sustained their shape and layered order, clearly seen from the cryo-cross-section (Figure 7A). All layers show clear borders and homogeneously distributed cells (Figure 7B). The top layer, representing the epidermis, was designed with a denser mesh and smaller pores to prevent the keratocytes from possibly migrating to other layers. Together with dECM, skin hydrogel represents the outer tissue barrier and keratocytes isolated from the skin, specifically the epidermis. For the middle layer, representing the dermis, we used dECM aorta hydrogel, rich in elastin, with a mesh and endothelial cells, representing small blood vessels. The bottom layer represented the subcutaneous layer, made from dECM LV hydrogel, as it had the highest stiffness, supporting the whole construct, together with a mesh and iASCs isolated from fat tissue.

We have successfully obtained a first-of-its-kind construct combining dECM with a PHBV+ P34HB scaffold. The biocompatible polymeric scaffolds have customised fibre diameters, organisation, and adjustable pore sizes. Still, their stiffness is closer to a plastic culture plate than the organ stiffness in the human body. On the other hand, dECM hydrogel stiffness is representative of organ stiffness [27], which makes it challenging to manipulate due to its fragility. It is a viscoelastic fluid at room temperature, filling in all pores once combined with the polymer. The dECM hydrogel provided a nutritional environment for the cells, supporting them in adhesion and survival [27]. The polymer provides support due to its stiffness and can be biodegraded; therefore, these two products complement each other, resulting in a biocompatible, easily personalised construct.

A variety of commercially available artificial tissues is available to patients: Biobrane^®^, Dermagraft^®^, Integra^®^, Apligraf^®^, MatriDerm^®^, OrCel^®^, Hyalomatrix^®^, and Renoskin^®^ [28,29]. Those are mostly one to two-layer models used predominantly for dressing burn wounds. Many more are still under research, with promising in vivo results showing improved wound healing, for example, with a PCL/SF nanocomposite fibrous construct [30], but again, aiming to represent only a single tissue layer. Full-thickness skin models have been reported in the literature, and some even implement nerve components with a silk-collagen-based polymer [31]. To our knowledge, none of the available tissue constructs could be biodegraded. A key property of our construct is its tuneable biodegradation.

We demonstrated the implementation of the dECM and the biodegradable polymer as an in vitro multilayered culture construct, representing three layers of human tissue: the epidermis, vascular layer, and subcutaneous tissue. The three-layer cell-seeded model sustained its composition and layered structure. We demonstrated the successful production and biocompatibility of this model with three different pre-seeded cell types. We represented the epidermis layer with skin ECM and keratocytes to represent tissue with high stiffness and barrier functions. The vascular layer comprises aorta ECM hydrogel and endothelial cells, representing highly vascularised tissue. The subcutaneous layer comprised left ventricle ECM and adipose-derived cells, representing soft, highly vascularised tissue.

Compared to other models, our model utilised animal-derived dECM hydrogels to more accurately depict the physiology of each layer and highlight the differences between them. This tuneable implant has diverse future implementations, ranging from simple burn wound coverage to diabetic ulcer regeneration patches, such as off-the-shelf full-thickness patches. It could be further personalised for individual patients’ use, such as being loaded with a needed drug or by using different organ-derived ECM hydrogels to resemble a different organ with its stiffness and composition. 

## 3. Conclusions

This study presented a first-of-its-kind bioengineered construct that integrates multiple-layered decellularised extracellular matrix (dECM) hydrogels with a 3D-printed biodegradable polymer scaffold (PHBV+P34HB) produced via melt electrowriting (MEW) technology to model an in vitro tissue graft. Our approach successfully combined the biological dECM with the structural precision and tunability of MEW-fabricated scaffolds. Among the various designs tested, a randomised fibre mesh proved to be the most favourable for supporting endothelial cell adhesion, highlighting the importance of microarchitecture in guiding cellular behaviour. Importantly, we demonstrated that the gradual degradation of the PHBV/P34HB mesh did not compromise cell viability, indicating good biocompatibility of the system over the culture period.

These findings confirm the potential of this hybrid platform as a promising tool for engineering vascularised soft tissue constructs. By providing both mechanical support and a bioactive matrix, the system establishes a foundation for the future development of functional tissue grafts, including more complex, multilayered tissue models and eventual in vivo translation.

## 4. Materials and Methods

### 4.1. ECM Hydrogel

Pig hearts were purchased from a slaughterhouse (Kroon Vlees, Groningen, The Netherlands). The aorta and left ventricles were extracted and dissected into 1 cm^3^ pieces. Ice-cold Dulbecco’s phosphate-buffered saline (DPBS) (Lonza Walkersville, Inc., Walkersville, MD, USA) was added, and a kitchen blender (Bourgini, Breda, The Netherlands) was used for further fractionating and mixing. The tissue homogenate was sonicated at 100% power for 1 min. The tissue was collected by centrifugation, washed twice with DPBS and incubated with 0.5% trypsin in DPBS (Thermo Fisher Scientific, Waltham, MA, USA) at 37 °C under continuous agitation for 4 h. Next, the suspension was washed twice with PBS and incubated overnight at 37 °C in Milli-Q^®^ water (Ultrapure water purification systems, Milli-Q EQ (Avantor, The Netherlands)) water under constant agitation. The tissue homogenate was incubated in excess saturated NaCl (6 M) for 3 h. Subsequently, it underwent multiple washes using PBS. The homogenate was incubated in a solution containing 1% SDS (Sigma-Aldrich, St. Louis, MO, USA), 1% Triton X-100, and 1% sodium deoxycholate (Sigma-Aldrich), with repeated PBS washes between each incubation step. To degrade any DNA present, the homogenate was treated with DNase for 24 h (30 µg/mL) (Roche Diagnostics GmbH, Mannheim, Germany) in the presence of 1.3 mM MgSO_4_ and 2 mM CaCl_2_, and the homogenate was washed six times with DPBS. Finally, the homogenate was collected after centrifugation, sterilised using 70% ethanol overnight, and washed before being stored in sterile MilliQ^®^ water at −20°C. The samples were then frozen rapidly using liquid nitrogen and subsequently subjected to freeze-drying using a FreeZone Plus freeze-dryer (Labconco, Kansas City, MO, USA). The freeze-dried samples were finely powdered using an Ultra-Turrax (IKA, Staufen, Germany). A concentration of 45 mg/mL ECM powder was digested using 2 mg/mL porcine pepsin (Sigma-Aldrich) in 0.01 M HCl at room temperature with continuous stirring for 6 h for LV and 24 h for aorta. After digestion, the ECM was neutralised by adding 1/10th volume of 0.1 M NaOH and then 1/10th volume of 10× DPBS to create an isotonic, pH-neutral ECM prequel. The hydrogels had a final concentration of 10 mg/mL of ECM.

### 4.2. Cell Culture

Immortalised Human Pulmonary Microvascular Endothelial Cells (HPMEC-ST1.6R, referred to in this manuscript as HPMEC) (kind gift of professor Kirkpatrick, University of Mainz, DOI: 10.1038/labinvest.3780385) were cultured with RPMI-1640 (Lonza, Basel, Switzerland) with 20% heat-inactivated fetal bovine serum (FBS) (Sigma-Aldrich), 1% penicillin/streptomycin (Gibco Invitrogen, Carlsbad, CA, USA), 1% L-glutamine (Lonza Biowhittaker, Verviers, Belgium), 5 U/mL heparin (LEO Laboratories Limited, Ballerup, Denmark), and 30 µg/mL of endothelial growth factors (home-made bovine brain extract). Immortalised human keratinocyte cell line (HaCaT, ATCC (LGC Standards, Molsheim, France)) and immortalised human adipose-derived stromal cells (iADSC13) [32] were cultured with Dulbecco’s Modified Eagle Medium (DMEM) (BioWhittaker, Walkersville, MD, USA) containing 20% FBS, 1 g/mL glucose, 1% penicillin/streptomycin and 1% L-glutamine. As the representative for dermal fibroblasts, spontaneously immortalised PK84 dermal fibroblasts [32] were used and cultured in the same medium as iADSC13, but with 10% FBS.

### 4.3. Live Staining

Live-dead staining with Hoechst nuclear dye (1:50 dilution, Thermo Fisher Scientific, Waltham, MA, USA) and calcein AM (1:200 dilution, Thermo Fisher Scientific, Waltham, MA, USA) was performed in FBS-free medium. Samples were incubated in the staining solution in the dark for 30 min at 37 °C, washed twice with PBS, and then imaged using an inverted fluorescence microscope (EVOS model M5000, Thermo Fisher). The following filters were used: DAPI (357/44 nm) and GFP (470/22 nm). At least three areas were randomly selected for each channel to capture images.

### 4.4. PHBV+P34HB Polymer Sourcing and Mesh Production

PHA filament used for 3D printing was sold under the commercial name of all PHA natural (ColorFabb, Belfeld, The Netherlands). The meshes were 3D printed via the MEW printer (MELT A-1204-0001-01D, Spraybase, Dublin, Ireland). The designs of the meshes consisted of parallel fibres with inter-fibre distances of 500 µm printed as continuous layers. The layers were positioned 90° from one another (square) or 30° from one another (rhombus). The number of layers varied between 7 and 8. The printing parameters were as follows: printing speed of 30–70 mm/s, nozzle diameter of 0.25 mm, collector-to-nozzle distance of 4 mm, temperature of 180 °C, voltage of 2.00–4.5 kv, and pressure of 0.050–0.425 bar. Depending on the degree of thermal degradation of PHBV+P34HB correlated to the printing time, the deposited fibres were either printed as straight lines (organised) or exhibited a “whipping” effect, during which the lines began to coil, resulting in randomised meshes. 

### 4.5. Degradation Enzyme Production

PHB depolymerase was produced using a recombinant *Bacillus subtilis* 168 strain. The *phaZ* gene from *Bacillus* sp. NRRL B-14911 [33], encoding a secreted PHB depolymerase, was genomically integrated using plasmid pDR111 (kind gift of Dr. D.Z. Rudner, Dept. Microbiology, Harvard Medical School, Boston, MA, USA) [34]. Gene expression is under the control of the IPTG-inducible hyper-spank promoter.

For impregnating MEW PHA meshes with PHA depolymerase enzymes, the spent supernatant of a liquid culture of this recombinant *B. subtilis* strain was used. The liquid culture was induced at an OD_600 nm_ of 0.4 with 0.1 mM IPTG for 16 h and centrifuged at 3320× *g* for 20 min. The supernatant was filter-sterilised using a Nalgene Rapid Flow 75 mm filter unit containing a 0.2 µm PES filter (595–4520, ThermoFisher Scientific, Breda, The Netherlands). the sterile supernatant was concentrated twofold by centrifuging at 3320× *g* using ultrafiltration tubes with a 10,000 MW cutoff (Pall, Fisher Scientific, Landgraaf, The Netherlands).

### 4.6. Polymer Meshes Characterisation

The pore size of MEW printed meshes was measured using light micrographs and ImageJ-1.54f densitometric software [35]. This was performed for all four different polymer designs used in the experiments: (1) organised square, (2) organised rhombus, (3) randomised, and (4) mixed organised/randomised. First, the scale was correctly set in ImageJ, and then 10 random pores were manually measured in each picture; for each design, 3 images were analysed (*n* = 3). The measurements of the pores were taken in two directions: length (L) (defined as the most extensive distance) and width (W), defined as the shorter distance, were plotted and analysed separately. All the pictures analysed were brightfield, taken with an inverted microscope (EVOS, ThermoFisher Scientific, Breda, The Netherlands), with 10× objective magnification. While we generated 2D images of a 3D object to assess pore sizes, only the distance between the fibres in the same 2D plane was determined (in µm).

### 4.7. Mechanical Characterisation of dECM Hydrogels and Composite Constructs

ECM hydrogels (aorta, left ventricle and skin) were produced as described in Section 4.1. The basic groups in this experiment were hydrogel with cells, hydrogel with mesh and cells, and mesh with cells. The cells used were HPMEC, as described in Section 2.2, and samples were incubated for 3 days before measurements were taken. 

To determine the viscoelastic properties of the organ-derived ECM hydrogels and hydrogel-mesh composites, low-load compression testing (LLCT) was used [36]. After placing the hydrogels on a standard microscope slide, they were compressed 20% for 1 s (strain rate, ε = 0.2). After this, the deformation was kept constant for 100 s while the force was monitored for the hydrogels. The hydrogels’ elastic modulus (stiffness (Pa)) was determined based on the stress-strain curves obtained during one second of deformation. The relaxation phase of the stress-strain curve was fitted to a generalised Maxwell equation (Figure 2A), yielding the time to 50% relaxation (s) and relative importance (%) of the contributing number of Maxwell elements. The relevance of the contribution was determined if the Maxwell element did not decrease the chi-square value by 3% compared to the previous element [37]. Three independent experiments were analysed using MatLab 2018 (MathWorks^®^ Inc., Natick, MA, USA). An outlier test was performed, and outlier values were removed.

### 4.8. Adhesion Assay

To prevent cell attachment to the well plate, low-adhesion 6-well plates (Corning^®^ Costar^®,^ Corning Inc., Corning, NY, USA) were used in all experiments where the polymer meshes were tested. Four different polymer designs were tested: (1) organised square, (2) organised rhombus, (3) randomised, and (4) mixed organised/randomised. The experiment was performed using two different cell types, separately: HPMEC and PK84 fibroblasts.

Using a scalpel, the polymer meshes were cut into pieces of approximately 1 cm^2^, sterilised by immersion in 70% ethanol for 24 h and washed afterwards with PBS. After air-drying, 600,000 cells per cm^2^ single mesh were added. For HPMEC, an RPMI-based medium was used; for PK84 DMEM, a high-glucose-based medium was used. The medium was refreshed every two days. Live-dead staining was performed on days one, three, and ten, and then images were obtained with EVOS (*n* = 3).

Picture analysis and cell count of alive cells were performed with ImageJ. All images were uniformly processed, and all were 10× magnified in the GFP channel (Calcein staining). First, background noise was removed, the rolling ball radius was set at 10 pixels, images were converted to binary, overlapping cells were separated by ‘watershed’, and only particles between 5 pixels^2^ and infinity were counted.

### 4.9. Composite Constructs of dECM Hydrogels and Randomised Polymer Meshes

Cells were mixed with pre-gel: 600,000 HPMEC were combined with 200 µL of the aorta, skin, and left ventricle pre-gel. Then, 1 cm^2^ of PHBV+P34HB meshes was immersed in the pre-gel liquid and incubated at 37 °C (*n* = 9) for gelation. Ultra-low attachment 6-well plates, except for the cell controls, were used to avoid cellular emigration and attachment to the plate. Live-dead staining was performed (following the procedure described in Section 4.3) on days one, four, and ten; samples were visualised with EVOS fluorescence microscopy. All images were uniformly processed and acquired with magnification in the GFP channel (calcein-AM staining). In ImageJ, background noise was removed, the rolling ball radius was set at 10 pixels, images were converted to binary, overlapping cells were separated by ‘watershed’, and only particles in the range between 5 pixels^2^ (500) and infinity were counted. At least three independent experiments were performed.

### 4.10. Enzymatic Degradation Studies

The PHBV+P34HB meshes were incubated in this sterile enzyme supernatant (as described in Section 4.5) for 24 h at 4 degrees before starting the experiment. The enzyme was removed, and the mesh was washed with PBS before the experiment began. Next, the constructs were composed as described in the previous section (*n* = 9). The medium was changed every two days. Live-dead staining was performed on days one, five, and 14; samples were visualised with EVOS. All images were uniformly processed, and all were 10× magnification in the DAPI channel (Hoechst nuclear staining). First, background noise was removed, the rolling ball radius was set at 10 pixels, images were converted to binary, overlapping cells were separated by ‘watershed, and only particles in the range between 5 pixels^2^ and infinity were counted.

Successful enzymatic degradation was defined as mesh polymer fibres breaking down and visually disappearing, resulting in lower total fibre length and width (mm).

### 4.11. Multi-Layered, Tissue-like Culturing Construct

Our multi-layered model consisted of three layers: 600,000 cells were mixed with dECM pre-gel solution for each layer, and a corresponding 1 cm^2^ polymer was embedded (covered from all sides) with the hydrogel-embedded cells (*n* = 9). The bottom layer comprised iADSC13 embedded in LV dECM hydrogel and supported by a randomised mesh. The middle layer comprises HPMEC embedded in aorta dECM hydrogel and supported by a randomised mesh. Finally, the top layer comprised HaCat keratinocytes embedded in skin dECM hydrogel and supported by a dense, randomised mesh with small pores. After mixing the components, each layer was incubated for 20 min before adding the next layer. Medium was added thirty minutes after the gelation of the top layer. In an earlier phase, we established that all three cell types could be efficiently maintained in a 1:1 (*v*/*v*) mixture of DMEM-based and RPMI-based medium, which was used for the duration of the experiment, changing it every three days. The experiment was conducted in low-attachment six-well plates. After 14 days, the constructs were washed with PBS and fixed with PFA. Cryosections of 10 µm were made and used for immunofluorescence and histochemical staining. This high section thickness proved essential to preserve the layer-like structure of the constructs.

### 4.12. Statistical Analysis

Statistical analyses were conducted using GraphPad Prism v9.2.0 (GraphPad Company, San Diego, CA, USA). The Shapiro-Wilk test was used first to check the normal distribution of our data. A 2-way ANOVA test was used with the experiment’s data to compare significant differences between groups and conditions. Tukey’s multiple comparisons test was used for group comparisons. A threshold of *p* < 0.05 was set for the corresponding statistical tests.

## Data Availability

Dataset available on request from the authors. Raw data includes a high number of microscopic imaging files.
